# Impact of small-scale irrigation on the livelihood and resilience of smallholder farmers against climate change stresses: Evidence from Kersa district, eastern Oromia, Ethiopia

**DOI:** 10.1016/j.heliyon.2023.e18976

**Published:** 2023-08-09

**Authors:** Ibsa Dawid Mume, Jema Haji Mohammed, Mohammed Aman Ogeto

**Affiliations:** a Oromia Agricultural Research Institute, Asella Agricultural Engineering Research Center, P.O.Box. 06, Asella, Ethiopia; b School of Agricultural Economics and Agribusiness, Haramaya University, P.O.Box. 138, Dire Dawa, Ethiopia; c School of Agricultural Economics and Agribusiness, Haramaya University, P.O.Box. 50, Dire Dawa, Ethiopia

**Keywords:** Climate change, Livelihood, Propensity score matching, Resilience, Small-scale irrigation, Ethiopia

## Abstract

This study mainly aimed to evaluate the impact of small-scale irrigation on the livelihood and resilience of farmers toward climate change in Kersa district of the eastern Oromia region of Ethiopia. A sample of 288 randomly selected households (158 non-adopters and 130 adopters) was used to gather the data. The data were analyzed using the resilience capacity index and propensity score matching methods. The resilience capacity index was utilized to summarize all the resilience indicators into a single value, and propensity score matching was used to evaluate the impact. The results of the average treatment effect on the treated analysis revealed that adopters were better-off in crop yields by 84.72 quintals per hectare, 55641.60 birr in total income, and by 2.02 resilience capacity index compared to non-adopters. The results of the study indicate that small-scale irrigation significantly improves farm households' livelihoods and mitigates the effects of climate change by enhancing their ability to respond to erratic weather events, which builds their resilience. Therefore, policymakers should prioritize small-scale irrigation practices to improve rural households' livelihoods and farmers’ resilience in areas with irregular rainfall and a high risk of drought.

## 1 Introduction

Climate change is arguably the most serious and complex challenge facing today's society, a crosscutting issue affecting several sectors and linked to other global issues [1]. Climate change is currently regarded as the most severe environmental threat, affecting a wide range of activities, especially in nations where agriculture is an important sector [2]. Developing nations are extremely exposed to climate change due to their economies' excessive reliance on climate-sensitive sectors [3,4]. Most smallholders rely on climate-sensitive rain-fed agriculture, and the sector is more susceptible to climate change effects than other sectors [5]. The negative consequences of climate change on agriculture are obvious, as its concerns influence the livelihoods of smallholder farmers [6].

Agricultural production systems that are predominantly rain-fed, use old technologies, and are operated by smallholder farmers are more sensitive to the adverse effects of climate change [7]. The livelihoods of smallholder farmers in Ethiopia remain seriously threatened by the adverse effects of climate change [8]. Increased climate variability (changing rainfall patterns, increased temperature, and lower precipitation) leads to more variability in production, causing low productivity and limited coping options for smallholder farmers [9]. The country is susceptible to the adverse effects of climate change due to its low level of economic development and reliance on rain-fed agriculture [10].

The Climate-Resilient Green Economy (CRGE) initiative was started by the Ethiopian government to protect the nation from the negative effects of climate change and to build a green economy that will assist Ethiopia in reaching its goal of becoming a middle-income nation by 2025 [11]. Improving agricultural production methods is one of the pillars of CRGE in order to attain food security and increase farmers’ incomes in order to improve resilient and climate change-adaptive systems. Adoption of climate-smart agriculture (CSA) practices is required to meet the aforementioned goals [12]. Adopting CSA practices can help farmers become more resilient to climatic variability and climate change [12,13]. In spite of any short-term difficulties, resilient households are those that effectively implement climate-smart agricultural methods and eventually escape poverty and vulnerability [14,15]. Improvements in farming systems through the adoption of CSA practices are essential to attaining climate resilience and enhancing sustainable livelihoods for farmers [16]. Small-scale irrigation is one of the most important CSA practices employed in developing countries as a climate change adaptation strategy for enhancing yields [17].

The usefulness of CSA practices in assisting farmers and their decisions about executing CSA practices and policies depend on their local resources, situations, and agro-ecology [18,19]. To combat the adverse implications of climate change on rural livelihoods, the adoption of CSA practices like the development of small-scale irrigation holds substantial promise for enhancing output and minimizing exposure to climate volatility [17,20].

Studies indicate the various roles of small-scale irrigation schemes on the livelihood of smallholder farmers, including diversification of crops, increased production, better income, and job opportunities [17,21]; ensuring food security and improved livelihoods [22]; stabilizing agricultural production and improving resilience [[23], [24], [25]]; coping with the negative effects of climate change by improving productivity and production volume [[26], [27], [28]]; and improving household income [[29], [30], [31]].

It is estimated that Ethiopia has an irrigation potential of 5,536,457 ha, of which 4,256,457 ha have been irrigated [32]. According to the authors, the Oromia region has plenty of water resources and irrigable land. The region has an irrigation potential of 1.7 million hectares of irrigable land, of which 1,350,000 ha are irrigated in different irrigation systems. In the study area, Kersa district has diverse watercourses that seem appropriate for small-scale irrigation farming. In the district, donors such as the International Fund for Agricultural Development (IFAD) were involved in irrigation scheme development and promotion to improve smallholder farmers’ livelihoods. More than 5071 ha of land in the district have the capacity to be irrigated, of which 2704 ha have been cultivated using various techniques of irrigation. A total of 5834 households in the district benefited from small-scale irrigation farming [33].

Despite this potential, there is little empirical evidence on how small-scale irrigation farming influences the livelihood and resilience of smallholder farmers to the effects of climate change in the study area. Thus, the purpose of this study was to assess how small-scale irrigation influenced the livelihood and resilience of farm households and to provide information for future investigations and policy interventions. Specifically, the study was established to address the following research questions: What aspects determine the use of small-scale irrigation? Does the use of small-scale irrigation enhance the livelihood and resilience of smallholder farmers against the adverse impacts of climate change?

## 2 Research methodology

### 2.1 Description of the study area

The study area is located in eastern Ethiopia, approximately 478 km east of Addis Ababa. Kersa is one of the districts in the Eastern Oromia region of Ethiopia. The estimated 199,601 people in the district are mostly rural (93.8%), with urban residents making up only 6.2% of the total population [33]. The district has a total population of 101,796 males and 97,805 females. The agro-ecology is classified as midland (74%), highland (20%), and some lowland (6%). The average annual rainfall is 886.5 mm, and the average annual temperature is 21.2 °C.

The crop-livestock production system is the main activity of the smallholder farmers in the district to improve their livelihoods. In order of importance, the most common cereal crops grown were sorghum, maize, wheat, barley, and pulses. *Khat* and vegetables are the known cash crops. Cattle, goats, and sheep are among the livestock species reared by the community in the district. The main economic activities were food crops, cash crops (*khat*), and livestock production. The most important crops sold were *khat*, potatoes, and onions. Land ownership, livestock production, and other resources determine the wealth status of the district [33,34].

### 2.2 Sources of data and methods of data collection

For this study, primary and secondary data sources were used. The primary data were collected using a semi-structured questionnaire at the household level through interviews. The secondary data were gathered from different documents, including official reports, published and unpublished articles, and other similar relevant documents from records of the district's agricultural development office and natural resources, to supplement the primary data.

### 2.3 Sampling procedures and sample size determination

A three-stage sampling approach was used to select the required sample households. First, Kersa district was purposefully selected based on its susceptibility to climate change and climate variability like erratic rainfall and drought [34], and the district has broad experience in implementing small-scale irrigation farming activities [13]. Secondly, four kebeles were selected randomly from 12 potential small-scale irrigation practices. Thirdly, the households were stratified into small-scale irrigation users and non-users. Finally, 288 representative sample households (130 adopters and 158 non-adopters) were selected using systematic random sampling techniques.

The proportional sampling approach was used to obtain the sample size from sample kebeles, which is calculated using the following [35] formula, and denoted as equation (1):

(1) $$n_{i}=\frac{\left(N_{i}\right)\left(n\right)}{\sum Ni}$$

Where, $n_{i}$- is the sample to be selected from the ith kebele, $N_{i}$ -is the total population living in the ith kebele. ∑Ni - Summation of population in four selected kebeles, n - total sample size for the district.

### 2.4 Methods of data analysis

#### 2.4.1 Descriptive statistical analysis

The descriptive statistics summarize household characteristics, while inferential statistics like the chi-square and *t*-test were used to check the significance of dummy and continuous variables. STATA version 17 was employed for the analysis.

#### 2.4.2 Measurement of households’ resilience to climate change

The estimation of the resilience of households to climate change followed an approach that was developed by Kathryn (2015), which is called the Resilience Capacity Index (RCI). The RCI compares household resilience in a systematic manner. It was used to summarize the different dimensions of resilience into a single number. Thus, since there are no well-defined weights assigned to the resilience indices, the Principal Component Analysis (PCA) was applied to attach relevant values to the different indices. The study used a two-stage method to estimate households’ resilience index, estimating five major resilience blocs using principal component analysis based on fifteen resilience indicators (Table 1) and computing the resilience index. The index was assessed based on the resilience blocs projected. This includes access to food and income, access to assets, good agricultural practices, stability, and adaptive capacity.

**Table 1:** Summary of resilience components and their indicators.

Resilience components	Indicators
Access to food and income (AFI)	Per-capita income of the households Food security status of the households
Access to assets (A)	Size of landholding Livestock ownership
Good agricultural practices (AP)	Amount of production Yields/productivity
Stability (S)	Drought existence Rainfall variability Livestock disease Crop failure Access to water Access to healthy
Adaptive capacity (AC)	Livelihood diversification Access to climate information Early warning system

In mathematical notation, the resilience capacity index is denoted as a function of the blocs as follows and is specified as in equation (2):

(2) RCI=f(AFI,A,AP,S,AC)

^w^here, RCI is the resilience capacity index, AFI is access to food and income, A is assets, AP is good agricultural practices, S is stability, and AC is adaptive capacity. Therefore, the resilience index is the weighted sum of the factors created and stated as in equation (3):

(3) $$RCI=\sum_{i=1}W_{j}F_{j}$$

^w^here, W_j_ is the weight of variable j and F_j_ is the factor under attention of variable j. The weights are the proportions of variance explained by each factor.

#### 2.4.3 Impact of small-scale irrigation on the livelihood and resilience of farm households

In the absence of baseline data utilizing observable variables, the propensity score is computed using a logit regression model to predict the average treatment effect of the outcome [36]. For this particular study, the outcome variables used for evaluation were annual income, yield, and resilience of households. The average change in the outcome variables was estimated using Propensity Score Matching (PSM). By comparing every individual observation from the treatment group with every individual observation from the control group that has identical observable characteristics, propensity score matching eliminates the possibility of self-selection bias [37]. In the binary treatment of the program, the treatment indicator $D_{i}$ equals 1 if individual i receives treatment, and 0 otherwise.

In this study, the treatment group denotes to households that participate in small-scale irrigation farming, while the control group is those that do not participate. The possible outcomes are then defined as $Y_{i}(D_{i}$) for each individual i, where i = 1, 2 … n, and then the treatment effect of individual i can be expressed as equation (4):

(4) $$Tᵢ=Y_{i}\left(D_{i}=1\right)-Y_{i}\left(D_{i}=0\right)$$

Where, Tᵢ is the treatment effect, $Y_{i}$ is the outcome on household i, and $D_{i}$ is a dummy indicating whether household i has received the treatment or not. However, it should be noted that $Y_{i}\left(D_{i}=1\right)$ and $Y_{i}\left(D_{i}=0\right)$ cannot be observed for the same household at the same time, and reliant on the position of the household in the treatment, either $Y_{i}\left(D_{i}=1\right)$ or $Y_{i}\left(D_{i}=0\right)$ is an unobserved outcome (counterfactual outcome). Because of this, it is impossible to quantify the individual treatment effect Tᵢ; instead we have to estimate the population average treatment effect. Hence, the average treatment effect on the treated (ATT) is specified as the following equation (5):

(5) ATT=E(Ƭ|D=1)=E[Y(1)|D=1]−E[Y(0)|D=1]

Thus, the counterfactual mean for those receiving treatment is denoted by -E[Y(0)|D=1], which is not observed. Following Caliendo and Kopeinig [38] and further process, we have the following expression as in equation (6):

(6) ATT=E[Y(1)|D=1]−E[Y(0)|D=0]=E[Y(0)|D=1]−E[Y(0)|D=0]

ATT is the so-called ‘self-selection bias; then, the true parameters of ATT are only identified if E[Y(0)|D=1]−E[Y(0)|D=0] = 0. By rearranging the equation above, equation (7) stated as follows:

(7) [Y(0)|D=1]−E[Y(0)|D=0]=0=ATT=E[Y(1)−Y(0)]

Common support region given by overlap 0 < p (D = 1/x) < 1. Ultimately, the general PSM model is specified as equation (8) as:

(8) $$Y_{ATT}^{PSM}=E_{P\left(X\right)|D=1}\;\left\{E\left[Y\;\left(1\right)\;|D=1,p\left(x\right)\right]-E\left[Y\;\left(0\right)\;|D=0,p\left(x\right)\right]\right\}$$

This suggests that the PSM estimator is the mean difference in outcomes over the common support region, suitably weighted by the participant's propensity score distribution. In this situation, propensity score matching solves the effect evaluation issue by generating control groups with propensity scores that are comparable to those of the treated. According to Caliendo and Kopeinig [38], there are six steps to implementing propensity score matching methods: estimating propensity scores, selecting the best matching algorithm, identifying common support region, checking the balance of covariates after matching, estimating the average treatment effects on the treated, and sensitivity analysis.

## 3 Results and discussion

### 3.1 Characteristics of sample households

The descriptive statistics suggest that men led the most households for both adopters and non-adopters of small-scale irrigation. The results indicated that households that adopted small-scale irrigation were more male-headed than female-headed. The results of the chi-square test for sex indicate that there was a statistically significant sex difference between the two groups at less than 5% probability level. Because female-headed individuals are more socioeconomically disadvantaged than male-headed individuals in many ways, they are slower to adopt small-scale irrigation activities, which require time, energy, and capital. The findings also revealed that farmers who adopted small-scale irrigation had more access to financial credit than non-adopters. The chi-square test result revealed that the two groups’ participation in off/non-farm activities was statistically different at a level of significance less than 10% (Table 2).

**Table 2:** Descriptive statistics of dummy variables.

Variables		Adopters (N = 130)		Non-adopters (N = 158)		Total (N = 288)		
	Dummy	Freq	%	Freq	%	Freq	%	P-value
Sex of HH	Female	10	7.70	25	15.82	35	12.15	
	male	120	92.30	133	84.18	253	87.85	0.036**
Access credit	Yes	43	33.08	31	19.62	74	25.70	
	No	87	66.92	127	80.38	214	74.3	0.009***
Off/non-farm	Yes	63	48.46	59	37.34	122	42.36	
	No	67	51.54	99	62.65	166	57.64	0.057*
Membership in cooperative	Yes	87	66.92	78	49.37	165	57.29	
	No	43	33.08	80	50.63	123	42.71	0.003***
Climate information	Yes	68	52.30	34	21.52	102	35.42	
	No	62	47.70	124	78.48	186	64.58	0.000***
Perception of climate change	Perceived	102	78.46	101	63.92	203	70.49	
	Not Perceived	28	21.54	57	36.08	85	29.51	0.007***

***, **, and * indicate significance at less than 1%, 5%, and 10% significance levels, respectively.

Adopters had higher rates of agricultural cooperative membership and access to weather information than non-adopters. This shows that small-scale irrigation practices are more likely to be used by farmers who are members of cooperatives and have better access to climate information in order to mitigate the impact of climate change on farm production. Moreover, the findings showed that adopters explicitly perceive climate change as being worse than non-adopters. This indicates that farmers who are more conscious of climate change are adopting small-scale irrigation practices as a climate change adaptation strategy to reduce the risks of climate change. The Pearson chi-square test also consolidates this result at less than 1% significance level (Table 2).

Furthermore, the mean age of adopters is lower than that of non-adopters, with a statistically significant difference between the two groups at the 5% probability level (Table 3). This implies that households headed by younger people are more likely to adopt small-scale irrigation than households headed by elders. Similarly, the results also revealed that households with a higher ratio of dependence and farming distance from irrigation water sources are less likely to engage in small-scale irrigation practices. Educational level, household size in adult equivalents, frequency of extension contact, and livestock ownership (TLU) were significant among adopters compared to non-adopters. This shows that farmers with better education, more household size and extension contact, and more ownership of livestock assets have a higher propensity to adopt small-scale irrigation to reduce the influence of climate change (Table 3). Households with large family sizes in adult equivalents are motivated to participate and more likely to implement small-scale irrigation farming than households with small family sizes since irrigation requires a larger labor force.

**Table 3:** Descriptive statistics of continuous variables.

Variables	Adopters (N = 130)		Non-adopters (N = 158)		Total (N = 288)		
	Mean	Std Dev	Mean	Std Dev	Mean	Std Dev	T-value
Age	39.75	7.36	41.81	6.77	40.88	7.10	2.46**
Education	6.07	3.62	4.72	3.42	5.33	3.57	−3.25***
Household size	6.39	1.72	5.97	1.37	6.16	1.55	−2.28**
Extension contacts	1.66	1.03	1.37	0.76	1.50	0.90	−2.71***
Livestock	3.27	1.43	2.52	1.71	2.86	1.63	−3.99***
Dependence ratio	0.73	0.30	0.84	0.40	0.79	0.36	2.60***
Market distance	0.95	0.38	0.98	0.30	0.97	0.34	0.78
Cultivated land	0.30	0.13	0.28	0.13	0.29	0.13	−1.24
Farm distance	0.58	0.15	0.61	0.08	0.60	0.12	1.92*
Farm experience	21.57	8.13	22.70	8.15	22.19	8.15	1.17

***, **, and * indicate significance at less than 1%, 5%, and 10% significance levels, respectively.

### 3.2 Measuring farm households’ resilience to climate change stresses

Small-scale irrigation practices adopters and non-adopters differed significantly in terms of resilience capacity index. Adopters witnessed more advantages in terms of having access to assets, good agricultural practices, access to food and income, stability, and adaptability (Table 4). All livelihood indicators significantly influence households’ resilience to climate change. This demonstrates that the improvement in the level of resilience is a function of all the resilience dimensions. The results also show that adopters of small-scale irrigation had better levels of resilience against the stress of climate change, but non-adopters had lower resilience. A study by Gutu [14] and Temesgen et al. [39] found similar results.

**Table 4:** Mean and standard deviation for households’ resilience and its components.

Variables	Adopters (N = 130)		Non-adopters (N = 158)		Total (N = 288)		
	Mean	Std. Dev	Mean	Std. Dev	Mean	Std. Dev	T-value
AFI	0.543	1.050	−0.447	0.689	2.81 × 10^−10^	1.00	9.60***
A	0.280	0.916	−0.230	1.00	−4.80 × 10^−09^	1.00	−4.46***
AP	0.926	0.757	−0.762	0.247	2.20 × 10^−10^	1.00	−26.37***
S	0.409	0.991	−0.336	0.875	1.03 × 10^−09^	1.00	−6.77***
AC	0.550	0.958	−0.452	0.784	1.12 × 10^−08^	1.00	−9.76***
RCI	1.280	0.895	−1.053	0.738	5.17 × 10^−09^	1.419	−24.2***

***indicates significance at less than 1% significance level.

Note:The primary characteristics of low resilience households (those with a negative mean value) were their increased vulnerability to the adverse effects of climate change and their diminished ability to mitigate the disruptive effects. They discovered that the losses caused by climate change provided little motivation to continue farming, prompting them to devote more effort to off-farm activities or move from rural to urban areas to increase their income and establish sustainable livelihoods. As a result, non-adopters have the lowest level of resilience in all selected parameters, especially agricultural production. Non-adopters have only one opportunity to produce crops through rainfall, whereas adopters produce crops two or three times per year. This suggests that non-adopters are more vulnerable to the effects of climate change. When compared to adopters, non-adopters were food insecure and had lower per capita income. Non-adopters have less access to land and livestock holdings in the study area. Non-adopters also had less access to climate information, did not diversify their livelihoods, and had less stability. They are not able to withstand the effects of climate change.

### 3.3 Impact of small-scale irrigation on yield, farm income and resilience

The PSM method and its procedural steps were used to evaluate the influence of small-scale irrigation practices on annual income, yield, and household resilience.

#### 3.3.1 Estimation of propensity scores

The propensity score estimate revealed that the model executed effectively with a chi-square value of 99.29 and a significant overall fitness at less than 1% (Table 5). Furthermore, the value of pseudo-R^2^ is lower (0.2504), showing that adopter households do not differ significantly from non-adopter households. Age, household size, credit access, frequency of extension contacts, livestock, dependency ratio, off/non-farm participation, cooperative membership, distance of irrigation, climate information, and perception of climate change had a significant influence on the propensity to participate in small-scale irrigation practices in the study area.

**Table 5:** Propensity score matching estimation (logit model).

Variables	Coefficient	Std. Err.	Z	P>|z|	M. effects (dy/dx)
Age of the HH	−0.070***	0.025	−2.77	0.006	−0.017
Sex of HH	0.193	0.480	0.40	0.687	0.047
Education of head	0.064	0.040	1.58	0.114	0.015
Household size	0.192**	0.097	1.98	0.048	0.047
Extension contact	0.382**	0.166	2.30	0.022	0.093
Access to credit	0.722**	0.340	2.12	0.034	0.178
Livestock holding	0.318***	0.095	3.32	0.001	0.078
Dependency ratio	−0.994**	0.410	−2.42	0.015	−0.244
Market distance	−0.212	0.440	−0.48	0.629	−0.052
Cultivated land	−0.196	1.104	−0.18	0.859	−0.048
Off/non-farm activity	0.618**	0.307	2.01	0.044	0.151
Membership in cooperative	0.902***	0.313	2.88	0.004	0.216
Distance from irrigation	−2.412**	1.217	−1.98	0.048	−0.593
Farmers' experience	−0.024	0.021	−1.14	0.255	−0.006
Climate information	1.085***	0.308	3.52	0.000	0.264
Perception of climate	1.101***	0.351	3.13	0.002	0.254
Constant	0.384	1.427	0.27	0.788	
Number of obs = 288			LR chi2 (16) = 99.29		
Prob > chi2 = 0.0000			Pseudo R2 = 0.2504		
Log likelihood = −148.61753			y = Pr (ACSSI) (predict) = 0.436		

**, and *** indicate significance at less than 5% and 1% significance levels, respectively.

#### 3.3.2 Distribution of propensity scores

**Fig. 1:**
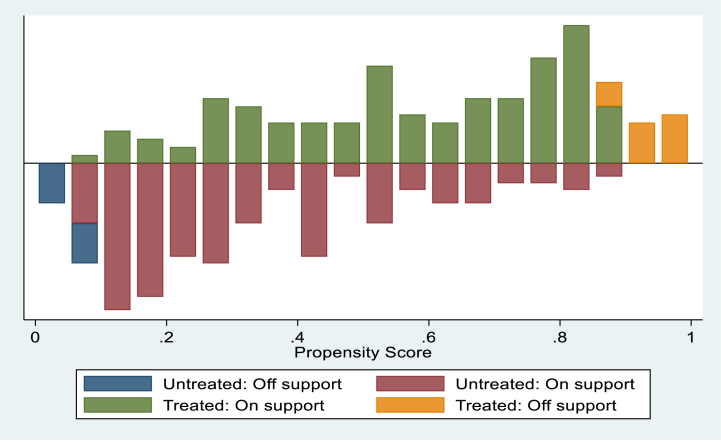
Propensity score distribution and common support region.

Propensity score matching matches each adopter based on a similar common characteristic with non-adopters. Hence, the distribution supports identifying the influence of small-scale irrigation adoption on household livelihood based on total income and yield of major crops and on the resilience of households. Fig. 1 displays the distribution of propensity scores and common support regions. The bottom halves of the histogram indicate the propensity score distribution of small-scale irrigation non-adopter households, and the upper halves depict the propensity score distribution of small-scale irrigation adopter households. The green colored (treated on support) and the red colored (untreated on support) indicate the observations in the adopters’ group and non-adopters that have an appropriate comparison, respectively, while the orange colored (treated off support) and the blue colored (untreated off support) indicate the observations in the adopters and non-adopters that do not have a suitable evaluation, respectively. The frequency of the propensity score distribution is shown on the y-axis.

#### 3.3.3 Identifying common support region

The assessed values of propensity scores for the sample households ranged from 0.014 to 0.984, with a mean score of 0.451. The propensity scores for adopters range from 0.072 to 0.984, with a mean score of 0.617. Similarly, the propensity scores for non-adopters range from 0.014 to 0.906, with a mean score of 0.315 (Table 6). The basic principle for describing the common support region is to delete all observations whose propensity score is smaller than the minimum propensity score of adopters and higher than the maximum of non-adopters [38]. Hence, the shared support region for both control and treated groups was found to be between 0.072 and 0.906. Having common support states that the two comparison groups can make a match. It implies that observations with propensity scores less than 0.072 and greater than 0.906 were discarded from the impact analysis. Therefore, 29 observations (15 from adopters and 14 from non-adopters) were cast off from impact analysis, and 259 sample households were recognized in the impact assessment procedure.

**Table 6:** Distribution of estimated propensity scores for sample households.

Group	Obs	Mean	Std. dev.	Min	Max	Cast off
Adopters	130	0.617	0.239	0.072	0.984	15
Non-adopters	158	0.315	0.226	0.014	0.906	14
Total households	288	0.451	0.276	0.014	0.984	29

#### 3.3.4 Choosing the best matching algorithm

To match adopters with non-adopter households in the common support region, various matching estimators were utilized. The decision on the ultimate choice of fitting matching estimator was based on four principles: equal mean test, smallest Pseudo R-square value, ATT result with the highest number of matched sample sizes, and insignificant likelihood ratio test. The suggestion is that a matching estimator that balances all covariates, a smaller Pseudo R-square value, a great matched sample size, and an insignificant LR chi-square are preferable. Caliper of radius 0.1 was found to be the best matching estimator since it had the least Pseudo R-square (0.010), insignificant LR chi-square (LR = 3.33, p = 1.000), and large matched sample size that were 115 treated and 144 control with a total of 259 sample households by discarding 29 unmatched from the total of 288 households.

#### 3.3.5 Matching quality

This test balances propensity scores and covariates to determine if there are any changes between conditions before and after matching conditioning. The results of the t-value showed that thirteen variables were statistically significant before matching; however, all the explanatory variables became statistically insignificant after matching, implying that matching helps to condense the bias related to observable features prior to matching. Moreover, the results revealed that the mean standardized bias for all the explanatory variables before matching was higher and in the range of 9.2%–57% in absolute value (Table 7). However, after matching, the mean standardized bias for all the covariates decreased and ranged from 0.9% to 11.2% in absolute value, which is smaller than the recommended value of 20% [37]. This indicates a high matching quality for the matching method. As a result, the treated and control groups had highly balanced covariates that were ready to be used in the ATT estimation process.

**Table 7:** Balancing test for covariates.

Variables	Before matching				After matching			
	Treated	Control	%bias	*t*-test	Treated	Control	%bias	*t*-test
Age of the HH	39.75	41.81	−29.1	−2.46	40.30	41.08	−11.0	−0.85
Sex of HH	0.923	0.841	25.4	2.11	0.922	0.917	1.6	0.14
Education of head	6.077	4.721	38.4	3.25	5.852	5.522	9.4	0.72
Household size	6.392	5.975	26.8	2.29	6.313	6.242	4.6	0.35
Extension contact	1.662	1.373	31.7	2.71	1.635	1.583	5.7	0.45
Access to credit	0.331	0.196	30.8	2.62	0.304	0.323	−4.3	−0.31
Livestock holding	3.279	2.524	47.7	3.99	3.116	3.17	−3.4	−0.24
Dependency ratio	0.736	0.848	−31.2	−2.60	0.755	0.732	6.4	0.50
Market distance	0.953	0.985	−9.2	−0.78	0.937	0.959	−6.2	−0.47
Cultivated land	0.302	0.282	14.7	1.24	0.298	0.295	2.5	0.17
Non/off-farm	0.485	0.373	22.5	1.91	0.435	0.458	−4.7	−0.35
Membership	0.669	0.494	36.0	3.03	0.635	0.627	1.5	0.12
Distance of irrigation	0.586	0.614	−22.2	−1.93	0.585	0.599	−11.2	−0.85
Farmers' experience	21.57	22.70	−13.9	−1.18	21.69	21.765	−0.9	−0.07
Climate information	0.477	0.215	57.0	4.86	0.435	0.430	1.0	0.07
Perception of climate	0.785	0.639	32.4	2.72	0.774	0.792	−4.1	−0.34

The joint significance test showed that the small Pseudo R-square and the insignificant likelihood ratio tests support the assumption that both groups have the same distribution of covariates after matching. The mean bias of the covariates was minimized, from 29.3% to 4.9%. The Beta was also minimized to 24.1%, which is less than 25% (Table 8). The results noticeably indicate that the matching technique is able to balance the features in the comparison of the treatment and control groups. Thus, it is used to compare observed treatment outcomes with those of a comparison group with common support to evaluate the impact of adopting small-scale irrigation between groups of households with comparable observed features.

**Table 8:** Tests for joint significance.

Sample	Ps R^2^	LR chi^2^	p > chi^2^	Mean Bias	Med Bias	B
Unmatched	0.253	100.19	0.00	29.3	29.9	129.6*
Matched	0.010	3.33	1.000	4.9	4.4	24.1

Note: * if B>25%, R outside [0.5; 2].

#### 3.3.6 Estimation of the average treatment effect on the treated (ATT)

For this study, the ATT is estimated based on the annual household income, yield, and resilience of smallholder farmers. According to the estimates of the caliper of radius matching, access to small-scale irrigation practices had a positive and significant influence on farmers’ annual total income, yield, and resilience. Specifically, the estimates showed that the implementation of small-scale irrigation practices significantly improved the agricultural crop yields, total annual income, and resilience of the adopters compared to their non-adopter counterparts.

The results of the impact analysis indicated that small-scale irrigation beneficiaries received an annual household total income of 90765.74 ETB per household, which is larger than that of non-adopters of small-scale irrigation with 35124.17 ETB annual income (Table 9). The findings indicated that, on average, adopting small-scale irrigation practices has increased annual households' total income by 55641.6 ETB for adopters compared to non-adopter households. The result also showed that there was a substantial difference between the adopters and non-adopters at less than 1% significance level in terms of household annual income. This suggests that small-scale irrigation has a positive influence on households’ earnings from both agricultural activities (cereals, livestock, vegetable, and *khat* production) and non-agricultural activities (off/non-farm) in the study area. This finding is similar to the discoveries of Leta et al. [29], Demsew and Ermias [30], Gadisa and Gebrerufael [31], and Abel [40], who show that small-scale irrigation has statistically significant impacts on household annual income.

**Table 9:** ATT estimation with Caliper of radius 0.1.

Outcome indicators	Sample	Treated	Controls	Difference	S.E	T-stat
Total income (ETB)	Unmatched	94199.59	28572.03	65627.57	4264.5	15.39^a^
	ATT	90765.74	35124.17	55641.58	5643.7	9.86^a^
Yield (Qt/ha)	Unmatched	120.07	34.74	85.33	3.97	21.51^a^
	ATT	118.71	33.99	84.72	5.27	16.06^a^
Resilience (RCI)	Unmatched	1.28	−1.05	2.33	0.09	24.24^a^
	ATT	1.22	−0.79	2.02	0.12	17.01^a^

a Indicates significance at less than 1% significance levels.

Similarly, the findings showed that the adoption of small-scale irrigation had a significant effect on the yields of major crops (vegetables and cereals) by households during the 2021/22 cropping season. The average yields of major crop production of adopters’ households were 118.71 Qt/ha and 33.99 Qt/ha for non-adopters (Table 9). This indicated that the mean impact of adopting small-scale irrigation practices on yield (output per hectare) for the aforementioned major crops was 84.72 Qt/ha. The result also showed that there was a substantial difference between the two groups at less than 1% significance level. This means that because of the adoption of small-scale irrigation, farmers improved their output by producing two or three times in a year using crop diversification and intensification. This finding is in line with the results of Kalkidan and Tewodros [26], Mengesha [27], Abel [40], and Adebayo et al. [41], who show that small-scale irrigation has a positive impact on the agricultural production and productivity of major farmers.

Furthermore, the implementation of small-scale irrigation practices had a significant influence on the resilience of households. As indicated, the resilience of adopter households was 1.22 RCI and −0.79 RCI for non-adopters. This showed that the mean impact of adopting small-scale irrigation on households’ resilience against climate change stresses by measuring it through different indicators and the capacity of farmers was 2.02 RCI (Table 9). The results also revealed that there was a major difference between the two groups in terms of the resilience of the households at less than 1% significance level. Thus, because of the adoption of small-scale irrigation practices as an adaptation approach to climate change, the adopters improved their resilience against climate change compared with non-adopters in the study area. This is in line with the findings of Abdissa et al. [23] and Menasbo [28], who show that small-scale irrigation practices improve the resilience of farm households against climate change pressures.

#### 3.3.7 Sensitivity analysis

The results of the Rosenbaum rbounds (rbounds) sensitivity analysis illustrated that the effects of the adoption of small-scale irrigation on outcome variables are insensitive to unobserved selection bias, even up to γ = 7, which is a very high value (Table 10). This implies that the study has taken into account significant covariates that affected both participation and outcome variables, as the p-critical values are significant for all outcome variables estimated at different levels of the critical value of gamma. As a result, the average treatment effects estimated on the yield, annual income, and resilience of households were extremely robust (insensitive) to the presence of unobserved features.

**Table 10:** Sensitivity analysis using Rosenbaum rbounds approach.

Outcome variables	e^γ^ = 1	e^γ^ = 2	e^γ^ = 3	e^γ^ = 4	e^γ^ = 5	e^γ^ = 6	e^γ^ = 7
Total income	0	2.6e-11	4.3e-08	1.8e-06	0.000	0.000	0.000
Yield	0	2.5e-11	4.1e-08	1.7e-06	0.000	0.000	0.000
Resilience	0	2.3e-11	3.9e-08	1.6e-06	0.000	0.000	0.000

Note: e^γ^ (gamma) = log odds of differential due to unobserved factors.

## 4 Conclusion and recommendations

The study was conducted with the major goal of evaluating the impacts of small-scale irrigation practices on the livelihood and resilience of smallholder farmers towards climate change stresses in Kersa district, eastern Oromia, Ethiopia. The findings showed that household size, extension contacts, credit service, livestock holding, off/non-farm activities, cooperative membership, climate information, and climate change perception had substantial positive relations with households’ adoption decisions. However, the results also showed that the age, dependency ratio, and distance of the farm from the irrigation source had a significant adverse relationship.

Based on a sample of matched treated and untreated groups, the influence of small-scale irrigation adoption on farm household livelihood and resilience was evaluated. The annual gross income, crop yield, and resilience of adopters and non-adopter households were compared using a propensity score matching method. According to the estimates of the caliper of radius 0.1, the implementation of small-scale irrigation practices had a positive and significant influence on the annual total income, yields of the main crops, and the resilience of households. This effect was evaluated as the average treatment effect on the treated. As a result, according to the estimations, adopting small-scale irrigation significantly improved agricultural yields and annual income, as well as the resilience of adopters compared to non-adopters.

Finally, the findings prove that small-scale irrigation practices assisted farmers in becoming resilient to the effects of climate change by increasing output and yields through crop diversification, which enhanced rural households’ standard of living. Adopting small-scale irrigation technology can therefore significantly improve livelihoods and abate the effects of climate change stresses by enhancing their capacity to respond to erratic rainfall events, which builds their resilience. Thus, to increase productivity and income and mitigate the hazards of climate change, the government and other concerned bodies should support small-scale irrigation practices. Moreover, to alleviate the effects of climate change pressures in the study area as well as the nation, the government and concerned stakeholders should seek to develop and support small-scale irrigation farming as an adaptation approach for climate change. This can be accomplished by offering appropriate extension services through development agents, providing training, and making credit available.

## Author contribution statement

Ibsa Dawid: Conceived and designed the study; Performed the research; Analyzed and interpreted the data and wrote the paper.

Jema Haji and Mohammed Aman: Contributed analysis tools or data.

## Data availability statement

Data will be made available on request.

### Additional information

No additional information is available for this paper.

## Declaration of competing interest

The authors declare that they have no known competing financial interests or personal relationships that could have appeared to influence the work reported in this paper.
