# Supplementary material for: Impact of small-scale irrigation on the livelihood and resilience of smallholder farmers against climate change stresses: Evidence from Kersa district, eastern Oromia, Ethiopia
Source: Heliyon. 2023 Aug 9;9(8):e18976. doi: 10.1016/j.heliyon.2023.e18976 (PMC10450858; doi:10.1016/j.heliyon.2023.e18976)
Supplement: Multimedia component 1 [file mmc1.docx]

**Appendix 1: Household Survey**

**Introduction:** This questionnaire is designed to analyze the Impact of small-scale irrigation on the livelihood and resilience of smallholder farmers against climate change stresses in Kersa district, Oromia Ethiopia. To identify factors that influence farmers’ decisions to adopt small-scale irrigation and to evaluate the impact of adopting small-scale irrigation scheme on the livelihood of farm households and improving their resilience against climate change stresses. We like to ensure that the results of the study will be only used for academic purposes and your response will remain confidential. The research team acknowledges your participation and genuine response to this survey.

**Part I: Supportive Information**

1. District: ______________ Kebele: __________________ Code:__________

2. Name of the household head_____________________________;

3. Name of the small-scale irrigation scheme;_________________________

4. Irrigation typology: 1. Modern. 2. Traditional

**Part II:**  **Questions on Household Demographic Characteristics**

1. Agro-ecological zones: 1. Highland 2. Mid high land 3. Lowland
2. Sex of the household head/respondent: 1. Male 0. Female
3. Category of the household head 1. Model 2. Middle level 3. Resource-poor
4. Age of the household head (in years)_____________
5. Marital status: a) Married: b) Single; c) divorced: d) widowed:
6. Educational status of household head in schooling year_________
7. Household size: No of Male ________ No of Female _____________Total ______
8. Which farming do you undertake? (a) irrigation farming (b) rainy season farming
9. Experience of household head in farming system (years) ______________
10. Experience of household head in small-scale irrigation practice (years) _________

**Part III: Landholding during 2021/22 G.C cropping season.**

1. Total cultivated land size in ____________________(ha)
2. Total cultivated land used for irrigation activity_________________(ha)
3. Land used for non-irrigable_____________________(ha)
4. Do you think that your piece of land is enough to support your family? 1. Yes 0.No
5. If No, what are the reasons? 1. Low soil fertility, 2. Small land size, 3. Large family
6. Did you shared-in/leased the land during 2013/14 E.C? 1. Yes 0. No
7. If yes, total land size______ ha; irrigated ______ ha, and rain-fed _______ ha.

**Part IV: Questions on Household Socio-economic Characteristics**

1. What farming systems you follow currently: i. Crop production only, ii. Livestock rearing only, iii. Mixed farming

2. What are the major crops grown? 1. Horticultural 2. Cereals 3. Khat 4. Mixed

**3. Major** **Crops production in 2021/22 G.C**. **cropping season**

3.1. Major crops grown under rain fed conditions

| No. | Crops grown | Area coverage(ha) | Purpose of growing | | | Total production (qt) |
| --- | --- | --- | --- | --- | --- | --- |
|  |  |  | For food | For market | Both |  |
| 1 | Sorghum |  |  |  |  |  |
| 2 | Maize |  |  |  |  |  |
| 3 | Wheat |  |  |  |  |  |
| 4 | Barley |  |  |  |  |  |
| 5 | Faba bean |  |  |  |  |  |
| 6 | Others (specify..) |  |  |  |  |  |

3.2. Major crops grown under irrigation farming

| No. | Crops grown | Area coverage(ha) | Total production | Purpose of growing | | |
| --- | --- | --- | --- | --- | --- | --- |
|  |  |  |  | Consumed (qt) | Sold (qt) | Both |
| 1 | Vegetables |  |  |  |  |  |
| 2 | Fruits |  |  |  |  |  |
| 3 | Khat (in birr) |  |  |  |  |  |
| 4 | Others |  |  |  |  |  |

**3.3. Income from Crop production**

3.3.1. Annual household income from cereals and fruits production during 2021/22

| Crop type | Cultivated land in (ha) | Total annual harvest(Qt) | Consumed  (Qt) | Sold(Qt) | Unit price(birr) | Total |
| --- | --- | --- | --- | --- | --- | --- |
| 1. **Cereals** |  |  |  |  |  |  |
| Sorghum |  |  |  |  |  |  |
| Maize |  |  |  |  |  |  |
| Chick pea |  |  |  |  |  |  |
| Others… |  |  |  |  |  |  |
| 1. **Fruit**s |  |  |  |  |  |  |
| Mango |  |  |  |  |  |  |
| Avocado |  |  |  |  |  |  |
| Orange |  |  |  |  |  |  |
| Papaya |  |  |  |  |  |  |
| (Others)… |  |  |  |  |  |  |
| 1. Khat |  |  |  |  |  |  |
| Total income |  |  |  |  |  |  |

3.3.2. Annual household income from irrigated vegetable crop production

| Crop type | Cultivated land in (ha) | Yield (Qt/kg) | Consumed  (Qt) | Sold(Qt) | Unit price (birr) | Total |
| --- | --- | --- | --- | --- | --- | --- |
| **Vegetables** |  |  |  |  |  |  |
| Onion |  |  |  |  |  |  |
| Tomato |  |  |  |  |  |  |
| Potato |  |  |  |  |  |  |
| Pepper |  |  |  |  |  |  |
| Cabbage |  |  |  |  |  |  |
| Other… |  |  |  |  |  |  |
| Tot. income |  |  |  |  |  |  |

4. Why do you select the above type of vegetables/crops for your irrigation farming? (1) Better price (2) good production (3) high disease tolerance (4) seed available (6) others__________________

**5. Livestock production in** 2021/22. **Cropping Season**

5.1. Number of livestock production and income generated from livestock products

| **No** | **Types of livestock** | **Total** | **Livestock sold** | **Unit price** | **Total sales price** | **Purpose** |
| --- | --- | --- | --- | --- | --- | --- |
| 1. | Cow(s) |  |  |  |  |  |
| 2 | Calves |  |  |  |  |  |
| 3 | Heifers |  |  |  |  |  |
| 4. | Oxen |  |  |  |  |  |
| 5. | Horses |  |  |  |  |  |
| 6. | Donkey |  |  |  |  |  |
| 7. | Mule |  |  |  |  |  |
| 8. | Goats |  |  |  |  |  |
| 9. | Sheep |  |  |  |  |  |
| 10. | Poultry |  |  |  |  |  |

| Type of products and by-products | Quantity | Unit | Amount  collected | Amount consumed  in a year | Sold in a year (birr) |
| --- | --- | --- | --- | --- | --- |
| Milk |  |  |  |  |  |
| Butter |  |  |  |  |  |
| Egg |  |  |  |  |  |
| Honey |  |  |  |  |  |
| Total income |  |  |  |  |  |

5.2. Income from the sales of livestock products and by-products

6. On average, how much income can you generate from your farming activities per year? Please Specify in Birr:

- 1. From grain crop products sales/crop production:___________________
  2. From selling livestock and livestock products:_____________
  3. From fruits product sales:_____________________
  4. From vegetables product sales:_____________________
  5. From khat product sales:_________________________

7. What is the major source of your income (MAP) ————? 1. Crop production, 2. Small ruminants, 3. Cattle production, 4. Fruit and vegetables, 5. Off/non-farming activities, 6. Beekeeping 7. Khat, 8. Others————————

8. Do you have any members of your family engaged in off/non-farm? 1. Yes 0. No

9. If yes, income generated from off/non-farm activities

| No | Off/non-farm activities | 1. Yes 0.No | Monthly income | Total annual income (in birr) |
| --- | --- | --- | --- | --- |
| 1 | Remittance |  |  |  |
| 2 | Khat trade |  |  |  |
| 3 | Petty trade |  |  |  |
| 4 | Civil servant |  |  |  |
| 5 | Income from rented land |  |  |  |
| 6 | Income from aid |  |  |  |
| 7 | Income from house rent |  |  |  |
| 8 | Income from wage/laborer |  |  |  |
| 9 | Fuel and charcoal sale |  |  |  |
| 10 | Livestock trading |  |  |  |
| 11 | Others (specify) |  |  |  |

10. On average how much is your total expenditure per year? Please specify in Birr____

**Part V: Questions on Institutional factors influencing adoption of SSI practice**

1. Distance of farmland from the irrigation water source in hours___________
2. Walking distance to the nearest main market in hr/min__________
3. Where do you sell the majority of your farm products? 1. on the farm, 2. at cooperative union shops, 3. at local markets, 4. Others ________________
4. Do you get market information about the price of inputs and outputs? 1. Yes 0. No
5. If yes, indicate the source of your information______________________________
6. Did you get a good price for your product? 1. Yes 0. No
7. If not, what are the reasons? 1. Low demand for the products, 2. Higher supply of the products, 3. Lak of information, 4. Lack of road access, 5. Others ___________
8. Do you have access to credit for your agricultural activities? 1. Yes 0. No
9. Have you received credit to finance your SSI activity for the last two years? 1. Yes 0. No
10. If yes, the amount you received in birr. —————, and sources———————
11. If not, what are your sources of finance to invest on SSI practices? 1. Own finance 2. No need to invest on SSI 3. Others, Specify, ————
12. If you did not use credits, what is your reason? 1. Lack of assets for collateral, 2. No one to give credit, 3. High-interest rate, 4. no need for credit, 5 = others
13. Is there a farmers’ training center (FTC) in your *kebele*? 1. Yes 0. No
14. How far is the FTC from your home? _________________________km.
15. Do you get advisory services from extension agents? 1. Yes 0. No
16. If yes, frequency of contact with DAs per month (in number) __________________
17. Has any member of the HH received advice on the adoption of SSI? 1. Yes 0. No
18. If yes, what kind of advisory services did you get (MRP)? 1. Ways to scale out SSI practice 2. Adoption of SSI to increase income and reduce poverty 3. Role of SSI practice to increase resilience 4. Awareness on the adoption of SSI practice 5. Other specify_____

**Part VI: Questions related with small-scale irrigation schemes practices**

1. Do you have access to small-scale irrigation practices? 1. Yes 0. No

2. If No, what were the reasons for not using irrigation? (1) No farmland in surface water access (2) No awareness about it (3) No capital (4) No labor (5) no irrigation infrastructure (6) Others______________

3. If yes, what is the size of the irrigable land__________________ ha?

4. When did you start using irrigation? ____________production year

5. How long do you use small-scale irrigation farming? _________years

6. What are your main objectives for doing irrigation farming? ___________________

7. What are the root causes to engage into small-scale irrigation? 1. Climate variability/ change, 2. Improved livelihood, 3. Only one production season and the production is not adequate, 4 others________________

8. Could SSI be one mechanism for climate adaptation? **_______________________**

9. Which SSI type do you use? 1. Modern scheme 2. Traditional river diversion 3. Motor pump 4. Treadle pump 5. others-

10. What is your source of irrigation water? 1. Modern river diversion irrigation scheme 2. Traditional river diversion irrigation scheme 3. Underground water. 4. Developed Spring. 5. If others, specify__________

11. Which type of crops did you mostly irrigate? a. Vegetables. b. Fruits c. Cereals d. Khat

12. How do you select type of crops for your irrigation farming? (a) Better price (b) Good production (c) Easy to cultivate (d) Seed availability (e) Others (Specify)___

13. How would you describe the yields of your crops? (Good, Average, Bad)__________

14. How many times do you produce per year using irrigation? ____________(number)

15. For what purpose do you use irrigation crop products? _______________________

16. Have you ever faced a problem of crop failure when using irrigation? 1. Yes 0. No

17. If yes, what was the reason? 1. Water shortage. 2. Waterlogging. 3. Poor administration of water distribution. 4. Others specify________________________

18. Do you think that irrigation has a positive effect on household livelihoods? 1. Yes 0. No

19. If yes, what is the positive effect of irrigation that you have been? 1. Diversification of crops grown. 2. increased agricultural production. 3. Increased household income. 4. Proper utilization of family labor.

20. If you wish to continue applying this practice, why? a. Risks are low b. Benefits are realized quickly c. Market is favorable d. Because of climate change and variability

21. Do you know the benefits associated with adoption of SSI practice? 1. Yes 0. No

22. If yes, what are the benefits? a. Increases productivity b. Enhances resilience to climate change c. Are efficient in the use of natural resources d. Enhances food security e. More profitable f. less costly

23. Before you joined irrigation farming/you were employed in the irrigation, what was the monthly estimated income of your household? __________________________

24. What is the estimated monthly income of your household now? _______________

25. Can you say you are better-off with your farming/ employment in the irrigation scheme than ever before? _______________________________

26. Kindly use the perception of small-scale irrigation schemes options below, to answer the following questions according to your level of agreement or disagreement: (1) Strongly Agree (2) Agree (3) I do not Know (4)Disagree (5) Strongly Disagree

|  | **Issue** | **Select** |
| --- | --- | --- |
| A | Since you have started using SSI, household income has increased |  |
| B | Since you have started irrigation your livelihood improved |  |
| C | Since you have started irrigation your resilience improved |  |
| D | Since you have started using SSI, your skills and knowledge have increased |  |
| E | Since you have started using SSI crop diversity has increased |  |
| F | Since you have started using SSI farm productivity has increased |  |
| G | Since you have started using small-scale irrigation your adaptive capacity has improved |  |
| H | Using small-scale irrigation requires more labor |  |
| I | Implementation of small-scale irrigation requires more skills |  |
| J | Using small-scale irrigation is costly to implement |  |

**Part VII: Questions related to Perception of climate variability, climate information, and the role of adopting SSI practice in building farmers’ resilience to climate change**

1. What is your perception of climate change in your kebele? 1. Changed. 2. Not changed. 3. I do not know
2. What do you think is the cause of climate change? 1. Human causes. 2. Natural causes. 3. Both. 4. Wrath of god, curse. 5. Others
3. Have you noticed any changes in temperature? 1. Yes. 0. No
4. If yes, please specify the trend of the change in temperature you have noticed?

1. Increasing. 2. Decreasing. 3. Similar. 4. Do not know

1. Have you observed climate variability in your area? 1. Yes 0. No
2. If yes, what changes have, you noticed. (MA) a. Drought b. Increased temperature c. Increased rainfall d. Increased frost e. Decreased rainfall
3. Have you ever suffered a significant loss of crop/livestock production due to climate variability? 1. Yes 0. No
4. If yes, what is the effect of climate variability on your crop/livestock production? (MA). a. decline in crop yields b. increased in death of livestock c. decline in livestock products d. increased weeds e. increased diseases f. decrease in soil fertility g. decrease in water availability.
5. Have you made any changes in your farming practices following the bad incidences of climate variability? 1. Yes 0. No
6. If yes, what kind of change or strategies you followed? ___________
7. What is the contribution of SSI practice in building Resilience of HHs

| No | Contributions of SSI practice | 1. Yes | 1. No |
| --- | --- | --- | --- |
| 1 | Increased yield and food security |  |  |
| 2 | Increased crops productivity |  |  |
| 3 | Improved livestock productivity |  |  |
| 4 | Reduced climate risk |  |  |
| 5 | Increased responsiveness to unpredictable weather patterns |  |  |
| 6 | Improved soil fertility status |  |  |
| 7 | Improved income and livelihood |  |  |

1. Does the adopted SSI have helped you to cope up with climate change? 1. Yes 0.No

13. Do you receive climate information? 1. Yes 0. No

14. If yes, what channels of communication do you receive this information.

| **Sources of information** |  | **Sources of information** |  |
| --- | --- | --- | --- |
| Radio and Television |  | Extension services |  |
| Newspapers |  | Neighbors and friends |  |
| SMS services |  | Indigenous forecasters |  |

1. If you have received climate information, are using the information? 1. Yes 0. No
2. If not, why? ____________________________________________________

**Part VIII: Questions related to Information on indicators of household resilience**

1. Absorptive capacity
   1. Do you have access to an early warning system to prepare yourself for the coming climatic shock? 1. Yes 0. No
   2. If yes who gives early warning to you? 1. Development agent 2. Metrological agency 3. Local NGO 4. If others specify__
   3. Are there mutual supporting practices that exist in your area during climatic shocks? 1. Yes 0. No
   4. If yes who support you to strengthen your ability to withstand climate variability? _____________________________________________
2. Adaptive capacity
   1. Are you satisfied with your agricultural production? 1. Yes 0. No
   2. Is agricultural production is enough for your consumption? 1. Yes 0. No
   3. Are you able to save any cash in the last 12 months to build resilience to climate variability? 1. Yes 0. No
   4. How your assets like land, livestock, saving and credit help you to build your resilience to climate. ____________________________
3. Transformative Capacity
   1. Are you diversifying your livelihoods? 1. Yes 0. No
   2. Have you accessed the social safety net to strengthen your long-term resilience to climatic shocks? 1. Yes 0. No
   3. If yes who provides the service for you? 1. Government 2. Private 3. NGO 4. Charity organization 5. others specify______
   4. Have you access drinking water? 1. Yes 2. No
   5. Who is the owner of these water sources? 1. Government 2. Private 3. Kebele/Community
   6. Do you get a sufficient amount of water from these sources? 1. Yes 0. No
   7. Is there conflict over the use of water? 1. Yes 0. No
   8. Do you have access to health services? 1. Yes 0. No

**Appendix 2: Checklist for Key Informants (KII)**

1. Do you understand climate change 1. Perceived 0. Not perceived

2. If you do not perceived climate change, why? ________________

3. If you perceived climate change, what practices used to adapt to climate change?

4. What are the climate variability indicators?

5. Do you face climate variability and climate change in your area?

6. What are the main impacts of climate change on your livelihoods?

7. How do you see SSI activities as a coping mechanism?

8. What is your view about the SSI practice?

9. What is your plan for scale-out the practice and building resilience to climate variability?

10. What are the major factors (challenges) hinder to scale-out SSI practice?

11. What are possible strategies to improve the existing problems?

12. How do you see the climate change adaptation differences between adopters and non-adopters of small-scale irrigation?

13. What was the role of stockholders (Government, NGO, Union, etc.) in SSI practice?

14. As an organization, are there provisions in the agriculture sector policies that support for innovation and adoption of small-scale irrigation practice? If yes in what way?

15. Have the extension services enhanced the adoption of SSI practice?

16. As an organization, have you ever funded the adoption of SSI by farmers? If not why?

17. If yes, has the funding enhanced the adoption of SSI practice among farmers?

18. Do you understand what climate information is all about?

19. Do you have access to receive the climate information? 1. Yes 0. No

20. Do you disseminate this information to farmers?

21. What channels do you use to disseminate the information?

**Appendix 3: Checklist for Focus Group Discussions (FGD)**

**A. Perception of climate change and its effects on farming, and climate information**

1. How do you perceive climate change in your kebele?
2. What are the indicators of climate variability in your kebele?
3. How do climate changes impose an impact on agricultural production?
4. What do you think the possible ways to minimize the impact of climate change?
5. What are the best coping strategies employed in your kebele?
6. Do you receive the climate information? If yes, where do you get this information?
7. In your own opinion, has this information influenced farmers to shift their practices to climate-smart agriculture practices such as SSI practice?

**B. Question-related to Small-scale irrigation practice and its contribution**

1. When adoption of SSI practice started in this village and how?
2. What types of irrigation methods are taking place in this kebele?
3. How do you start irrigated agriculture?
4. What motivates to adopt SSI?
5. Could small-scale irrigation be one mechanism for climate adaptation?
6. What do you think about adoption of SSI practice? Is it necessary to build resilience to climate variability? How?
7. What is your general opinion on the role of SSI on improving resilience of farmers?
8. What benefit do you obtain from small-scale irrigation?
9. How do you see irrigated agriculture with other livelihood activities?
10. Is there any difference in income between irrigators and non-irrigator? What is the difference between these two groups?
11. Why other farmers in the kebele do not adopt SSI?
12. What are the major types of horticultural crops produced under SSI in this area?
13. For what purpose do you use irrigation crop products?
14. What are the major factors face in adopting the SSI practice in your area?
15. What are the expectations from different organizations in relation to SSI practice?
